# Growth Performance, Serum Biochemical Indices, Duodenal Histomorphology, and Cecal Microbiota of Broiler Chickens Fed on Diets Supplemented with Cinnamon Bark Powder at Prestarter and Starter Phases

**DOI:** 10.3390/ani11010094

**Published:** 2021-01-06

**Authors:** Mohammed M. Qaid, Saud I. Al-Mufarrej, Mahmoud M. Azzam, Maged A. Al-Garadi, Hani H. Albaadani, Ibrahim A. Alhidary, Riyadh S. Aljumaah

**Affiliations:** 1 Department of Animal Production, College of Food and Agriculture Sciences, King Saud University, Riyadh 1145, Saudi Arabia; salmufarrej@ksu.edu.sa (S.I.A.-M.); mazzam@ksu.edu.sa (M.M.A.); malgaradi@ksu.edu.sa (M.A.A.-G.); hanee7811@gmail.com (H.H.A.); ialhidary@ksu.edu.sa (I.A.A.); rjumaah@ksu.edu.sa (R.S.A.); 2 Veterinary Medicine Department, Faculty of Agriculture and Veterinary Medicine, Thamar University, Dhamar 13020, Yemen

**Keywords:** broiler chicks, cinnamon, goblet cell, growth promoter, triiodothyronine

## Abstract

**Simple Summary:**

During the first days of chicks’ lives, chicks are still developing the thermoregulatory system, the gut function, and the immune system. The aim of this study was to investigate the effects of supplemental *Cinnamomum verum* as a phytogenic feed additive in the starter diet. Our findings suggested that the addition of *Cinnamomum verum* at 2000 mg/kg might increase body weight gain over 1–10, 1–14, and 1–21 days of age by improving relative breast weight, elevating serum albumin concentrations, duodenal villus height, and goblet cell density at prestarter period.

**Abstract:**

Ross 308 broiler chicks (*n* = 240) aged 1 day were assigned to five groups for eight replicates (six chicks for each) (3♂ and 3♀). Basal dietary groups were supplemented by 2000, 4000, and 6000 mg/kg cinnamon (CN) for 21 days. Basal diet alone was used as a negative control, and basal antibiotic diet (Colimox) was used as a positive control. At 10, 14, and 21 days of age, chicks that received 2000 mg CN and Colimox had a higher body weight, resulting in an increase in body weight gain. CN also resulted in the maximum improvement in the feed conversion ratio and feed efficiency over 1–21 days at the level of 2000 mg/kg. At days 10, the maximum relative breast weight was 2000 mg/kg of CN. Mean serum albumin concentrations, duodenal villus height, and goblet cell density increased (*p* < 0.05) by 2000 mg/kg of CN, and mean serum globulin and total protein concentrations and crypt depth increased (*p* < 0.05) by 6000 mg/kg of CN compared with control. Increased cecal Escherichia coli number was CN dose-dependent. In conclusion, dietary inclusion of 2000 mg/kg CN can be applied as an alternative to in-feed antibiotics for broiler starter diet.

## 1. Introduction

Several studies, organizations [1], and firms are attempting to produce poultry with the highest genetic potential, manufacture simple diets free from or containing fewer pharmaceutical products, and reduce the impact of poultry products on human health [2] either by law [3] or by voluntary removal by egg and meat producers. Antibiotics used in human medicine, agriculture, and veterinary medicine and in the diets of broilers and animals are used as growth promoters to maximize feed potency and control pathogens [4,5]. However, the use of antibiotics in poultry and livestock is associated with an increase in multidrug-resistant germs [6]. Furthermore, [7] reported that antibiotics used in birds on the first day of age have negative effects on microbial colonization and intestinal function over a 14-day period. The prohibition of antibiotic use in farm animals has encouraged researchers to explore alternative agents such as enzymes, organic acids, and phytogenic feed additives such as spices and herb plants and products derived from them [8,9].

Phytogenic feed additives are used in broiler feeds, and their effective economic impact on body weight (BW) and feed conversion ratio (FCR) is highly distinct throughout the first stages of life [10,11]. During the initial days of life, the bird’s thermoregulation, immunological, and intestinal systems do not appear to be fully developed and slightly decrease in functions until age 7–10 d post-hatching [12,13]. Studies have reported that the development of the gastrointestinal tract, particularly the intestinal mucosa, immune competence, thermoregulatory function, and intestinal microbiome is affected by the nutrient source and nutritional management during the prestarter phase [14,15]. A healthy intestinal microbiota has favorable effects on the availability of essential trace elements, which is beneficial for the synthesis of thyroid hormones [16]. Moreover, the initial starter diet is one among the windows of chance for the supply of appropriate nutrition with alternative nonantibiotic products to broiler chickens, such as microbiome and natural herbs [17].

Cinnamon is a natural spice obtained from the inner bark of cinnamon tree. This tree is distributed throughout the world and includes approximately 250 species [18]. Cinnamon bark may be important as a phytogenic feed additive within the starter diet because it contains several phytochemicals such as cinnamaldehyde, eugenol, camphor, caryophyllene, plenty of water, and fat-soluble vitamins and also carbohydrates, proteins, fats, several minerals, and electrolytes, in addition to phytonutrients such as β-carotene, β-cryptoxanthin, lutein, zeaxanthin, and lycopene [19]. Consequently, cinnamon possesses antiviral [20], antimicrobial [21], antioxidant [22], antidiabetic, and antihypercholesterolemic effects [23]. Thus, it has been reported that cinnamon may be used as a natural food product preservative [24] and appetite stimulant, aids in digestion to secrete digestive enzymes, and can, hence, improve the performance of broilers [25].

We found several studies in the literature that have investigated the effect of cinnamon powder supplementation on broiler performance, carcass traits, and meat acceptability; however, the doses used varied considerably, ranging from 0.2 to 70 g (i.e., 0.02% to 7%), and the results were not consistent [10,26,27,28]. Any dietary phytogenic supplementation to the basal diet that may be expected to improve the performance and/or health of birds with the lowest dose and the lowest cost and without side effects may be referred to as reasonable doses. Therefore, we selected reasonable doses of cinnamon to check for varying doses in previous studies and determine the best trend for these doses. Hence, the aim of this study was to evaluate the effects of the inclusion of cinnamon as in-feed antibiotic substitution on the growth performance, digestive organ development, serum thyroid hormones, and selected cecal microflora of broilers fed a broiler diet from 0 to 21 days of age.

## 2. Materials and Methods

### 2.1. Proximate Analysis of Cinnamon Bark Meal

Dry cinnamon bark was obtained from a neighborhood store in Riyadh, the capital of Saudi Arabia, and then shipped to the Department of Animal Production, King Saud University. Upon arrival, the bark was crushed and ground using a blender to a fine powder (particle size: 0.25–0.30 mm). The cinnamon bark powder was analyzed directly and mixed with dietary groups. The water content of the dried grounded cinnamon bark was evaluated using a drying oven (Binder, Bohemia, NY, USA). The crude protein level was determined by the Kjeldahl technique (N X 6.25) using a 2020 Digester and a Velp UDK 140 distillation unit. The raw fat content was evaluated using the Soxhlet apparatus. The raw fiber content was determined using a Dosi-Fiber. The ash content was analyzed by incinerating the dried samples at 600 °C for 6 h. The acid detergent fiber content was determined based on [29] methods nos. 930.15, 990.03, 920.39, 978.10, 942.05, and 973.18, respectively, as described by [30]. The neutral detergent fiber content was analyzed as described by [31]. Gross energy (kcal/kg) was calculated using a bomb calorimeter. All data were expressed on the basis of dry matter.

### 2.2. Phytochemical Analysis of Cinnamon Bark Meal

The dried cinnamon bark powder (20 g) was macerated with 200 mL of 50% methanol at 25 °C for 24 h, filtered using a filter paper, and centrifuged at 4000× *g* for 30 min. Methanol was evaporated by a rotary evaporator at 40 °C, as described by [32]. Next, 10 μL of supernatant was injected into a high-performance liquid chromatography (HPLC) column. The analysis was conducted using Agilent Technologies’ series HPLC equipped with Zorbax RP-C_18_ column (1.0 cm long × 4.6 mm i.d., Agilent Shimadzu, Kyoto, Japan). Caffeine, catechin, gallic acid, and chlorogenic acid were used as external standards for detecting the contents of alkaloids, flavonoids, phenols, and polyphenols, respectively. Detection was performed by measuring the ultraviolet absorbance at 280 nm. The mobile phase was flowed at a rate of 1 mL/min and consisted of water, acetonitrile, ethyl acetate, methanol, and glacial acetic acid (89:6:3:1:1 v/v/v/v/v). All chromatographic analyses were performed at 25 °C ± 2 °C.

The cinnamon bark meal extract was evaluated by gas chromatography-mass spectrometry (GC-MS) on an Agilent (Palo Alto, CA, USA) 6890N gas chromatograph equipped with an Agilent HP-5MS column (30 m × 0.25 mm × 0.25 μm film thickness) and a 5973N selective mass detector. The oven temperature was increased from 60 °C to 320 °C from 2 to 20 min at a rate of 6 °C/min. The chemical constituents were successfully identified by comparing their mass spectrums, retention indices, and bioactive compound quality with those of the database library of National Institute of Standards and Technology (NIST-based AMDIS software).

### 2.3. Bird Husbandry, Dietary Treatments, and Experimental Design

The experiment was conducted at the Animal Production Experimental Farm of King Saud University (KSU), located at Riyadh, Saudi Arabia. The experimental procedures complied with the King Saudi Arabia standards on animal use (KSU-SE-20-44) and were approved by the local animal care and welfare committee of King Saud University. The study site is located at a longitude of 46.72° E, a latitude of 24.92° N, and an altitude of 612 m, with a mean temperature of 26.4 °C and rainfall (annual precipitation) of 110.6 mm, and has a semi-warm sub-humid climate [33].

A total of 240 as-hatched 1-day-old broiler chicks (Ross 308) were obtained from a commercial hatchery and brought to the research unit. All birds were vaccinated at the hatchery against infectious bronchitis, Newcastle disease, and infectious bursal disease virus. Upon delivery, the chicks were sexed by feather sexing, weighed individually, and assigned to five treatments. Each treatment included eight replicate cages with six broiler chickens (3♂ & 3♀) per replicate cage based on a completely randomized block design. The chickens were reared under the same management conditions in an environmentally controlled house in a cage measuring 58 cm in length, 50 cm in width, and 35 cm in height. The relative humidity and temperatures were 65% and 33 °C up to 5 days of age and were decreased gradually to 50% and 24 °C at 21 days of age, respectively. A photoperiod was used as a continuous lighting program (23L:1D) “23 h on:1 h off”.

The treatments consisted of a basal diet (negative control), a basal diet supplemented with 2000, 4000, and 6000 mg/kg CN bark meal, and basal diet supplemented with colimox (a mixture of amoxicillin and colistin antibiotics at 200 and 150 mg/kg, respectively) (positive control) on top of feed from 1 to 21 days of age. The supplemented levels used in the present study were based on the Maximum Ingredient level Optimization Workbook (MIOW) [34]. The experimental diets either fulfilled or exceeded the National Research Council [35] requirements as appropriate. Chickens were fed mash diets with ad libitum access to feed and water (nipple drinkers). Basal dietary ingredients were obtained separately from the commercial feed company “ARASCO,” Riyadh, Saudi Arabia. The basal diet was then analyzed, formulated, and mixed in a mashed form with a corn/soy-based basal diet. The ingredients and nutrient levels of the basal diet are presented in Table 1.

### 2.4. Growth Performance Indices

BW was determined at 1, 7, 10, 14, and 21 days of age per replicate. At age 1 d, the dietary treatments were divided into 40 pails (1 pail/replicate) and weighed individually (feed given). Diets were placed at the feeders routinely. At age 7, 10, 14, and 21 days, the leftover of the diets at the feeder and buckets were weighed to calculate feed intake (FI) per replicate cage as follows: FI = sum of feed offered—Feed leftover at the weighing time. FCR was calculated as FI divided by body weight gain (BWG). Feed efficiency (FE) was calculated as BWG divided by FI.

### 2.5. Samplings

Birds were weighed individually before they were slaughtered at age 10 days. All samples were collected from a single female bird per replicate (*n* = 8 birds; one chick/replicate). Blood samples were collected from the wing vein, and then serum was separated by centrifugation (3000× *g*, 10 min). The serum was aspirated by pipette and stored in Eppendorf tubes at −20 °C until analyses. Birds were humanely slaughtered using a sharp knife by cutting through the jugular vein, carotid artery, and windpipe. The empty weight of the proventriculus and gizzard and the weight of whole heart, spleen, liver, pancreas, thymus, and bursa of Fabricius were recorded. Furthermore, whole skinless breast meat with keel bone and whole legs (thighs with drumsticks) were weighed and expressed as g/100 g of live BW. In addition, 1 g of cecal contents was collected and stored at −80 °C until processed. Finally, 1 cm of the medial portion of duodenum was taken, washed in a saline physiological solution, and then fixed in 10% buffered formalin.

### 2.6. Serum Biochemical Indices

Tests for liver function and enzymatic activities were conducted to evaluate the levels of certain proteins and enzymes in serum, including total protein, albumin, uric acid, and hepatic transaminase enzymes, primarily aspartate aminotransferase (AST) and alanine aminotransferase (ALT), which were determined using commercial diagnostic kits according to the manufacturer’s instructions (Randox Laboratories Limited Ltd., Crumlin, UK) via a spectrophotometric analyzer (Randox, Rx, Crumlin, UK). Total thyroid hormones T3 (Triiodothyronine) and T4 (Thyroxine) in serum were analyzed using a T3 and T4 ELISA Kit (RecombiLISA Total Triiodothyronine (T3) ELISA (E1010) and RecombiLISA Total Thyroxine (T4) ELISA (E1020) (MDSS GmbH Schiffgraben 41, 30175 Hannover, Germany) using a microplate reader (MR-96A; Mindray Bio-Medical Electronics Co., Ltd., Shenzhen, China).

### 2.7. Morphometric Analysis of Duodenum

For enteric morphometric analysis, 1-cm segment of the duodenal midpoint was removed from each euthanized bird, washed in a physiological saline solution, and fixed in 10% buffered formalin for 72 h. Each segment was then embedded in paraffin, and 20 cross sections measuring 2 μm in thickness of each sample were cut. Each of the five semi-serial cuts was placed on a single microscopic slide and stained with Alcian blue and periodic acid-Schiff (PAS) reagents. Next, 10 well-oriented villi were selected to evaluate the duodenal morphology using the Olympus DP72 microscope. Morphological parameters, including villus height, villus base, villus surface area, lamina propria thickness, and crypt depth, were estimated. The height of the villus was measured from the top of the villus to the villus crypt junction (top of the lamina propria) of each villus. Crypt depth was measured based on the distance from the junction to the basement membrane of the epithelial cells at the bottom of each crypt [37]. The ratio between villus height and crypt depth was then calculated. The villus width was measured at the base area of each villus. The villus surface area was calculated using the following formula: 2π ∗ (VW/2) ∗ VL, where VW = villus width and VL = villus length [38], which were measured using the Nikon microscope (Nikon Corp., Tokyo, Japan) and Olympus digital video camera (DP72, Aartselaar, Antwerp, Belgium) equipped with the cell Sens software package. Goblet cells along the 10 oriented villi were visually counted under 200× magnification of the microscope.

### 2.8. Cecal Microflora

First, 1 g of cecal content was diluted in 9 mL of buffered peptone water and then the suspension was serially diluted 10-fold. From each tube, 0.1 mL was transferred to each of the different selective media. *Lactobacillus* spp. were enumerated on MRS agar. The medium was incubated at 37 °C with 5% CO_2_ in an anaerobic incubator (Thermo Fisher Heracell150, Waltham, MA, USA) for 72 h. *Escherichia coli* (*E. coli*) and *Salmonella* spp. were enumerated by culturing on MacConkey agar and Salmonella Shigella agar at 37 °C for 24 h, respectively. All the enumeration data were expressed as colony-forming units (CFU) (Log_10_ CFU/g of excreta).

### 2.9. Statistical Analysis

The General Liner Models (GLM) of the Statistical Analysis System [39] statistically analyzes data collected by one-way ANOVA for growth performance and physiological indices, and the results are expressed as mean ± SEM. Each replicate cage represented an experimental unit for growth performance indices (replicate cages, *n* = 8 per treatment). For samples, one female broiler was slaughtered per replicate. Duncan’s multiple range test was used to compare mean values, and the differences between mean values were considered to be significant at *p* < 0.05.

The model equation is described as follows:

(1) $$\gamma_{ij}=\mu +T_{i}+e_{ij}$$

where *γ_ij_* is the individual observation, μ is the general experimental mean, *T_i_* is the effect of the *i*th treatment, and *e_ij_* is the random error normally distributed with mean zero and variance σ^2^ ε ijk~N (0, σ^2^).

## 3. Results

### 3.1. Proximate Analysis and Phytochemicals of Cinnamon Bark Meal

The results of proximate analysis of the chemical composition of cinnamon (CN) bark meal are presented in Table 2.

**Figure 1:**
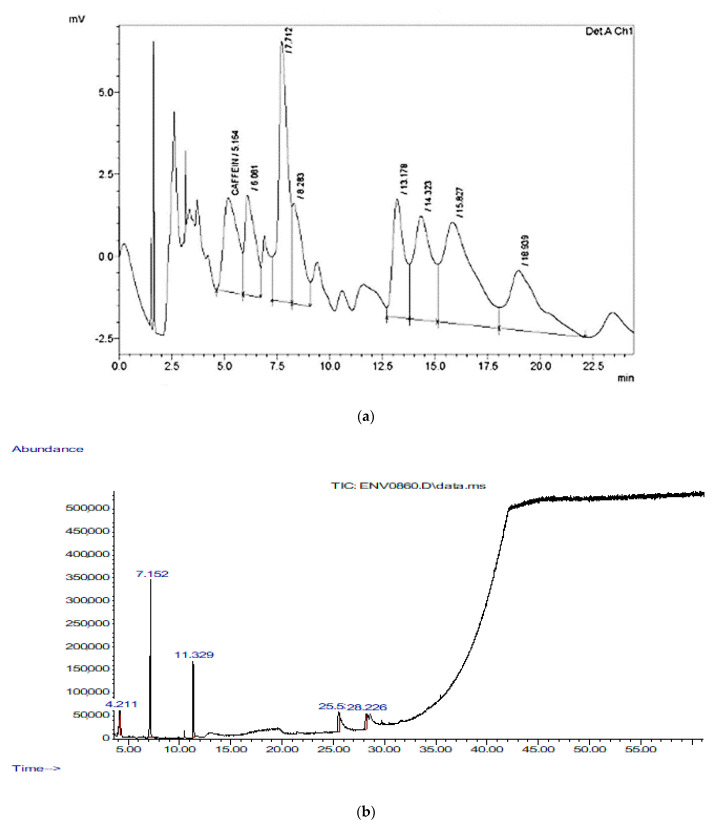
(**a**) HPLC chromatogram of standard of flavonoids and phenol mixture at 280 nm of cinnamon; (**b**) GC-MS tracing of the bark extracts of cinnamon.

Caffeine was measured by HPLC, and its concentration was 700 μg/g with a retention time of 5.164 min, an area of 132,732, and a height of 2865. In addition, the HPLC test detected another unknown component that was larger than caffeine, with a height of 7915, a retention time of 7.712 min, and an area of 242,491 (Figure 1a). Therefore, GC-MS was used to detect the major volatile compounds of cinnamon bark extracts. A total of 26 different volatile compounds were identified, as shown in Figure 1b and Table 3. Cinnamaldehyde, (E)-2-propenal, 3-phenyl-, hexadecanoic acid, methyl ester, pentadecanoic acid, 14-methyl-, methyl ester, oxime-, methoxy-phenyl-, and 2-methyl-benzofuran were the compounds with the highest quality detected by GC-MS in the CN bark extract.

### 3.2. Growth Performance Indices

The effects of experimental treatments on average BW, BWG, FI, FCR, and FE of broiler chickens are presented in Table 4 and Table 5. At age 10, 14, and 21 days, 2000 mg/kg of CN feed improved the average body weight (ABW) compared with negative control (238.20, 410.60, and 850.67 g vs. 224.00, 375.36, and 780.47 g; *p* = 0.039, *p* = 0.003, and *p* = 0.005; respectively). Fortunately, from 14 to 21 days of age, feeding with 2000 mg/kg cinnamon with addition of competitive antibiotics resulted in a nonsignificant gradual increase in the ABW of birds. During 1–10, 1–14, and 1–21 days of age, the 2000 mg/kg of CN feed improved the BWG compared with negative control (22.43, 29.32, and 37.71 vs. 20.63, 26.36, and 34.24; *p* = 0.037, *p* = 0.004, and *p* = 0.003, respectively). Fortunately, the BWG of chicken fed 2000 mg/kg of cinnamon was gradually and nonsignificantly increased, with the birds showing an increase in lifespan, compared with chicken fed with the antibiotic.

Food consumption, FCR, and FE of broiler chickens were not significantly (*p* > 0.05) affected by the dietary treatments at 1–7, 1–10, 1–14, and 1–21 days. However, during 1–21 d, the FCR of chickens in the 2000 mg/kg cinnamon group was significantly better (*p* = 0.027) than that of chickens in the negative control group (0.97 vs. 1.05). Moreover, during 1–21 days, feeding with 2000 mg/kg cinnamon significantly (*p* = 0.026) improved the FE of broiler chickens compared with negative control (1.04 vs. 0.95).

### 3.3. Serum Biochemical Indices

**Figure 2:**
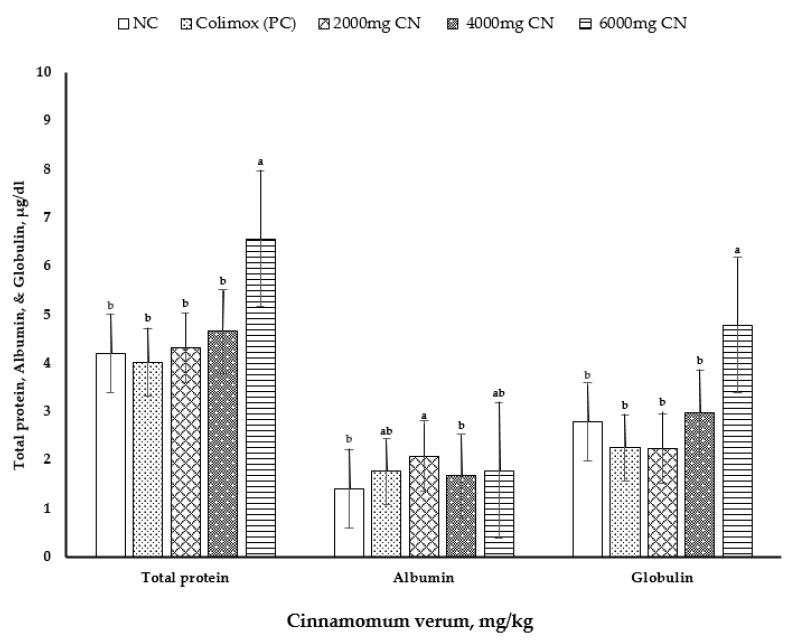
Effect of dietary treatments on serum levels of total protein, albumin, and globulin. Values are mean ± standard error (SE), *n* = 8 chicks per treatment (1 bird/replicate). NC: negative control group = commercial feed, no additives by cinnamon powder; PC: positive control group = commercial feed, treated by antibiotic Colimox powder (mixture of amoxicillin and colistin at 200 and 150 mg/kg, respectively); 2000, 4000, and 6000 mg/kg treated groups = commercial feed, treated by *Cinnamon verum* powder. ^a,b^ It indicates significant differences (*p* < 0.05) in serum total protein, albumin, and globulin compared with each CN level to the non-supplemented control diet.

The effects of cinnamon bark meal (CN) on serum constituent indices at 10 days of age are shown in Table 6 and Figure 2. At 10 d of age, the concentrations of serum total protein (*p* < 0.006) and globulin (*p* < 0.007) were significantly higher with the addition of CN at 6000 mg/kg than those in other treatments (Figure 2). Moreover, the highest serum albumin levels (*p* < 0.013) were observed with CN supplementation at 2000 mg/kg, and the lowest levels were observed with CN supplementation at 4000 mg/kg and with negative control diets. However, serum uric acid level, AST and ALT activities, triiodothyronine hormone (T3) level, and thyroxine (T4) level showed no differences among the dietary treatments (*p* > 0.05).

### 3.4. Organ Development

The effects of CN bark meal on the relative weights of developing organs at 10 d of age are shown in Table 7. Addition of CN bark meal at 2000, 4000, and 6000 mg/kg resulted in increased breast meat (% BW) compared with negative control diet (*p* = 0.0113), but there was no difference between them or when compared with the positive control diet. As shown in Table 7, no marked distinctions in the heart, liver, proventriculus, gizzard, pancreatic and lymphoid organs, bursa of Fabricius, spleen, and thymus could be observed (*p* > 0.05).

### 3.5. Morphometric Analysis of Duodenum

Table 8 summarizes the effect of treatments on duodenal morphology at 10 days of age. Broiler chickens receiving 2000 mg/kg of cinnamon had a significantly greater duodenal villus height (*p* < 0.01), villus height:crypt depth (*p* = 0.024), goblet cell count (*p* < 0.0001), and goblet cell density per 100 μm villus area (GCD/100 µm villus area) (*p* < 0.0001) than the antibiotic and control groups. The dietary supplementation of CN bark at different levels resulted in a decrease in the width of duodenal villus compared with the negative control group (*p* < 0.0001), but there was no difference when compared with the antibiotic group. Birds receiving 2000 mg/kg control diet had a larger villus surface area than those receiving antibiotics and 2000 mg/kg of cinnamon (*p* < 0.01), but the values were similar to those of chickens receiving 2000 and 4000 mg/kg of cinnamon. As shown in Table 8, the intestinal morphology of chickens was positively affected by the addition of CN bark in the starter diet.

### 3.6. Cecal Microflora

The effects of cinnamon bark meal (CN) on selected cecal microflora, viz., *Lactobacillus*, *E. coli*, *Salmonella*, and aerobic bacteria, at 10 days of age are summarized in Table 9. Feeding a diet with cinnamon increased (*p* = 0.012) the number of cecal *E. coli* and decreased the *Lactobacillus*-to-*E. coli* ratio compared to those obtained with positive and negative control diets. However, when compared between the cinnamon groups, the cecal *E. coli* count decreased numerically with an increase in the cinnamon level, and the *Lactobacillus*-to-*E. coli* ratio increased with an increase in the cinnamon level. The number of aerobic, *Salmonella* spp., and *Lactobacillus* spp. remained unchanged among the dietary treatments.

## 4. Discussion

Colimox is a combination of amoxicillin and colistin and was used in this study based on the study of [40]. They reported that Colimox has considerable value when used in broiler chickens for controlling necrotic enteritis compared with use of amoxicillin or colistin separately. Besides antibiotics, herbs and other phytogenic products could also control and limit the colonization of numerous pathogenic bacteria in the chicken gut, leading to greater efficiency in the utilization of food and resulting in enhanced growth and improved FE [41]. The external standards used in this work in HPLC have antimicrobials, antioxidants, and anti-inflammatory agents [42,43]. In this study, only caffeine and seven unknown components were detected in the CN extract by HPLC. This finding has been supported by [44], which also detected caffeine in cinnamon bark oleoresin microcapsules using acetone extraction. Therefore, GC-MS was used to identify the unknown components, which detected cinnamaldehyde as major active polyphenol component and 25 volatile compounds in the methanolic CN bark extract (Table 3). This finding is supported by [45], which also detected most of these identified volatile components. In [46], the authors reported that cinnamon has high levels of cinnamaldehyde, followed by eugenol and carvacrol. Moreover, these bioactive compounds are known to exert antioxidant effects [22,47,48]. Birds of 10-days age were selected for slaughter and detected a variety of physiological indicators, as the first 10 days of age (prestarter period) is considered the critical time of a broiler chick’s life. Small intestinal mucosal function, thermoregulatory system, and the immune system are undeveloped, immature, and still undergoing maturation up to 7–10 days of age. On the other hand, the main aim of the starter diet is to ensure proper nutrition (ingredients, nutrients, and feed additives) in order to overcome possible stress during the early post-hatch period. Here, therefore, the broilers’ performance was recorded and calculated at 7, 14, or 21 days of age in order to choose the best periods of age that cinnamon could improve performance when adding to the starter diet. At the prestarter and starter phases of age in the present study, the 2000 mg/kg of CN feed improved the BW of chickens. Furthermore, the positive impact of cinnamon additives on the digestive system and nutrient absorption was more pronounced at younger ages, because the BW, BWG, and breast weight (% BW) increased, which then improved the FCR and FE in chickens fed 2000 mg/kg of cinnamon diet in the starter period (1–21 days). There is a possibility that it will be better if the concentration is lower. In addition, here, we tracked the addition of CN at 2000 mg/kg on day 1, 7, 10, 14, 21, and found an increase in body weight gain over 1–10, 1–14, and 1–21 days of age. In the poultry sector, a good start has resulted in a good finish, but a bad start has not been recoverable or compensated. Thus, we have predicted that the positive effect of CN at 2000 mg/kg on body weight gain up to 21 days of age may be continuous until the age of slaughter. Similar to our results, Ref. [10] suggested that dietary inclusion of 2 g/kg cinnamon can be used in broiler diets as an alternative to antibiotics based on their results of increased BW, reduced FCR, and unaffected FI of broilers at 28 d of age. In [28], the authors also reported that chickens fed with cinnamon powder at levels of 250, 500, 750, and 1000 ppm showed a markedly higher live BWG and improved FCR compared with the control group during the first 3 wk, but their FI was decreased. That result was consistent with the findings of [49], which reported that supplementation of cinnamon oil at 100- and 200-ppm levels in the diet had a positive effect on the performance of broilers. Furthermore, Ref. [50] described that the use of cinnamon powder at levels of 500 and 1000 mg/kg may have a positive effect on broiler performance compared with control. However, Ref. [51] did not observe any significant impact on the performance of female broilers fed on diets supplemented with cinnamaldehyde. Similarly, Ref. [52] suggested that cinnamon infusions did not favor the performance of broilers. Nevertheless, the addition of a phytogenic blend of Piper betle, P. nigrum, Aerva lanata, and Cynodon dactylon was found to increase BW, coinciding with an increase in the duodenal villus height and villus height:crypt depth without affecting FI [53], which was similar to our finding with chickens fed a diet with 2000 mg/kg of CN. Cinnamon contributed as a phytogenic blend through one or more active compounds such as some flavonoids with medicinal importance and phenols with known antioxidant properties [53,54,55]. A larger surface area enhances nutrient digestion and assimilation and consequently maximizes BW [56]. Villus height and crypt depth are considered as indicators of good development of the intestine [57]. In normal conditions, the intestine presents a better villus height:crypt depth ratio. In particular, in the present study, the best villus height:crypt depth ratio was found in chickens that received 2000 mg/kg of CN. Cinnamon has a more bitter taste and an intense spicy smell, which may affect appetite and acceptability to FI. Consistent with [58], the use of CN bark powder, irrespective of its level, did not affect FI (*p* > 0.05), probably due to the better tolerance of broiler chickens to its smell and taste (slight citrus notes and sweet, spicy, hot fragrance). Consistent with [10], the experimental treatments had no effect on the relative weights of internal organs.

The early post-hatch nutrition probably plays a major role in the growth and regeneration of skeletal muscle by stimulating satellite cells in postnatal growth in livestock [59] and broiler chicks [60]. Here, an enhancement in BW and breast weight did not involve an increase in serum T3 hormone levels in the cinnamon-fed groups. Conversely, Ref. [61] and [62] reported a positive correlation between body growth and the increase in T3 hormone levels in birds, which could improve the ability of neonatal chicks to control body temperature and then increase BWG.

Albumin is the first of several proteins produced by the liver. The body requires albumin and total protein to fight against infection and exert other functions; when their levels are lower than normal, liver damage or disease may occur. In this context, Ref. [49] reported that cinnamon increases the level of serum albumin. In contrast, Ref. [10] reported that the inclusion of cinnamon did not affect serum albumin. The binding site of cinnamaldehyde and its major metabolite bound to serum albumin site I and site II, respectively [63]. Albumin, transthyretin, and thyroxine-binding globulin (globulin protein) are three essential thyroid hormone-binding proteins in circulation [64]. Thyroxine (T4) bound to these three binding proteins in the blood stream at around 10%, 15%, and 75%, respectively, and 0.03% of T4 is free [65]. Therefore, alterations in the synthesis of globulin or albumin in the liver could affect the total circulating thyroid hormone levels. The increase in serum albumin level may be associated with the improvement of protein synthesis and amino acid transport, which coincides with less oxidative stress and toxicity [66]. Khafaji et al. observed that the use of cinnamon powder could have a positive effect on certain physiological traits by significantly increasing the levels of total protein, globulin, T3, and T4 in groups fed 500 and 1000 mg/kg of CN compared with control group [50].

The enzymes ALT and AST are generally found in low levels in the bloodstream, and their levels increase when liver or muscle damage occurs. Hence, they are considered to be safety indicators and can reflect the degree of liver damage [67,68]. In the present study, the additives did not induce any adverse effects on the safety profiles of chickens. Choct et al. reported that the duodenum could be selected for morphology measurements because the height of the villus in the duodenum reached a peak between 6 and 8 d post-hatching, whereas the height of the villus in the jejunum and ileum increased after 10 days of age [69]. Moreover, Reynolds et al. described that the duodenum is the first part of the small intestine facing the microbiota that penetrates through the oral route. Goblet cells secrete mucin 2, which is a major constituent of the mucus that lines the bowel tract and generates a protective barrier between pathogens and epithelial intestinal cells and is, therefore, important for the health of chickens [70].

The addition of CN 2000 mg/kg resulted in an increase in the goblet cell count. In the present study, an obvious increase in crypt depth was detected with increased cinnamon levels. The increase in crypt depth may be favorable due to an increase in the number of proliferating stem cells, which could maximize the count of mucin-producing goblet cells [71]. Feeding a diet with 6000 mg/kg CN decreased the duodenal villus height, villus width, villus surface area, goblet cell count, and villus height-to-crypt depth ratio. This finding may be due to the irritation of intestinal tissues [72]. Our data are in agreement with the findings of [73], which reported that villus height and villus height-to-crypt depth ratio in the duodenum were increased by dietary cinnamon essential oil supplementation. Surprisingly, feeding a diet with 6000 mg/kg of CN decreased the cecal *E. coli* count; therefore, all chickens had a healthy status, with no mortality being recorded. Moreover, feed conversion and FE were not decreased in chickens fed a diet with 6000 mg/kg of CN. Reduced growth performance is a sign of pathological gut inflammation [74,75]. Dietary cinnamon essential oil was found to reduce the prececal *E. coli* count [73].

In addition, [75] reported that there were fewer changes in microbial populations in the large intestine of animals than those in the small intestine due to their competition with the host for nutrients [76]. Moreover, *E. coli* is considered to be a part of the cecal microbiome and may become a pathogenic agent due to stress, poor welfare, host factors, or as a secondary infection [77]. Furthermore, *E. coli* may function as a reservoir for T3 by binding it to bacterial thyroid-binding hormone [78]. In the present study, the addition of CN (2000–6000 mg/kg) resulted in nonsignificantly elevated serum triiodothyronine levels. Therefore, a shorter villus may not highly correlate with the BW of broiler chickens during the starter phase, and vice versa. It appears plausible to measure the levels of cecal short-chain fatty acids (acetate, propionate, and butyrate) and cecal function in the future study to evaluate the protective effect of CN on *E. coli* infection. Previous studies, on the other hand, indicated that CN had antimicrobial activity. Here, it was found that cinnamon at levels of 2000–6000 mg/kg increased *E. coli* colonies compared to negative and positive diets, which could be attributed to either cinnamon, considered to be an enhancer of *E. coli* growth, or the doses tested, considered to be below the threshold of cinnamon activity against *E. coli* colonies.

## 5. Conclusions

The demand for natural phytogenic feed additives would increase when the use of antibiotics is completely prohibited. Based on the results of our study, it can be concluded that cinnamon may improve digestion and absorption. In particular, dietary supplementation of 2000 mg/kg of cinnamon produced satisfactory results and can be considered to be beneficial as a phytogenic feed additive growth promoter for broilers fed a corn–soybean meal basal diet. This benefit can be attributed to its combined positive effects on BWG, FCR, FE, villus height, villus surface area, goblet cell count, and serum albumin level during the starter period. Moreover, feeding 6000 mg/kg of cinnamon had positive effects on total protein and globulin levels and crypt depth. Further study is required to elucidate the molecular mechanism of cinnamon bark.

## Figures and Tables

**Table 1:** Ingredients and nutrient level of control feed at starter phase, 1–21 days.

Ingredients, %	1–21 days
Yellow corn, 7.8%	53.22
Soybean meal, 45%	37.85
Wheat bran	2.00
Corn gluten meal	1.40
Rice bran oil	1.50
Di-calcium phosphate	1.98
Limestone	0.90
Sodium chloride (NaCl)	0.40
DL-methionine	0.29
l-lysine HCL	0.21
Choline chloride	0.05
Vitamin- mineral premix ^1^	0.20
Cinnamon bark ^2^	0.00
Total	100
Nutrient content (%, based on as-fed basis) ^3^	
Crude protein	22.80
Digestible lysine	1.26
Digestible sulfur amino acids	0.91
Digestible threonine	0.77
Calcium	0.93
Non-phytate P	0.45
Metabolizable energy, kcal/kg	2900

^1^ Vitamin-mineral premix comprises the following per kg: vitamin B1, 3200 mg; vitamin B2, 8600 mg; vitamin B3, 65,000 mg; pantothenic acid, 20,000 mg; vitamin B6, 4300 mg; biotin 220 mg; antioxidant “butylated hydroxyanisole (BHA) + butylated hydroxytoluene (BHT)”, 50,000 mg; B9, 2200 mg; B12, 17 mg; vitamin A, 12,000,000 international unit (IU); vitamin D3, 5,000,000 IU; vitamin E, 80,000 IU; vitamin K3, 3200 mg; copper, 16,000 mg; iron, 20,000 mg; iodine, 1250 mg; manganese, 120,000 mg; zinc, 110,000 mg; and selenium, 300 m. ^2^ The basal diet supplemented with 0, 2000, 4000, and 6000 mg/kg cinnamon; and Colimox antibiotic powder (mixture of amoxicillin and colistin at 200 and 150 mg/kg, respectively) on top of feed; supplemented levels were recommended based on the Maximum Ingredient level Optimization Workbook (MIOW) [34]. ^3^ Calculated based on to [36].

**Table 2:** Proximate analysis of cinnamon bark meal ^1^.

Item	% (on a Dry Matter Basis)
Moisture (% as fed)	10.29
Dry matter (% as fed)	89.71
Crude protein	4.43
Ether extract (lipid)	4.03
Ash	3.18
Total crude fiber	24.35
^2^ Nitrogen-free extract (NFE)	53.72
^3^ Organic matter	96.82
Total fiber fractions:	
A. Acid detergent fiber	45.75
B. Neutral detergent fiber	65.34
Gross Energy (kcal/kg)	4974.52

^1^ Chemical composition analysis conducted in duplicate; ^2^ NFE (calculated by difference) = 100-(Moisture + protein + lipid + ash + fiber); ^3^ Organic matter = 100-Ash.

**Table 3:** Major volatile compounds of the methanolic extract of cinnamon bark meal ^1^.

RT(min)	Bioactive Chemical Constituents	Quality	MW (amu)	Molecular Formula
4.108	4-Ethylbenzoic acid, 2-butyl ester	78	206.131	C_6_H_18_O_3_Si_3_
4.108	2-Amino-6-methylbenzoic acid; 2-Amino-4-methylbenzoic acid	59	151.063	C_8_H_9_NO_2_
4.211	Oxime-, methoxy-phenyl-_	87	151.063	C_8_H_9_NO_2_
4.211	4-Ethylbenzoic acid, hexyl ester	50	234.162	C_15_H_22_O_2_
7.152	Hexamethyl cyclotrisiloxane	56	222.056	C_6_H_18_O_3_Si_3_
7.152	Silane, 1,4-phenylenebis[trimethyl-	50	222.126	C_14_H_26_Si_2_
11.329	Cinnamaldehyde, (E)- 2-Propenal, 3-phenyl-	97	132.058	C_9_H_7_ClO
11.329	Cinnamaldehyde, (E)- 3-Phenyl-2-propyn-1-ol	91	132.058	C_9_H_8_O
11.329	2-methyl-Benzofuran	87	132.058	C_9_H_8_O
11.329	Benzene, (2-propynyloxy)-	72	132.058	C_9_H_8_O
11.329	Benzenemethanol,.alpha.-ethynyl-	64	132.058	C_9_H_8_O
11.329	Benzopyrimidine, 3,4-dihydro- or 3,4-Dihydroquinazoline	59	132.069	C_8_H_8_N_2_
11.329	2-Propenal, 3-phenyl-	58	132.058	C_9_H_8_O
11.329	2-Propenoyl chloride, 3-phenyl-, (E)- (Cinnamoyl chloride)	53	166.019	C_9_H_7_ClO
11.329	4-Methylcoumarine-7-cinnamate	53	306.089	C_19_H_14_O_4_
11.329	2-Nitrophenyl cinnamamide	50	268.085	C_16_H_14_N_2_O_4_
11.329	3-Butenoic acid, 2-oxo-4-phenyl-	50	176.047	C_10_H_8_O_3_
25.536	Hexadecanoic acid, methyl ester	99	270.256	C_17_H_34_O_2_
25.536	Pentadecanoic acid, 14-methyl-, methyl ester	98	270.256	C_17_H_34_O_2_
25.536	Tridecanoic acid, methyl ester	83	228.209	C_14_H_28_O_2_
25.536	Hexadecanoic acid, 2-methyl-	76	270.256	C_17_H_34_O_2_
25.536	Nonadecanoic acid, methyl ester	70	312.303	C_20_H_40_O_2_
25.536	Octadecanoic acid, methyl ester	62	298.287	C_19_H_38_O_2_
25.536	Methyl tetradecanoate	58	242.225	C_15_H_30_O_2_
25.536	Pentadecanoic acid, methyl ester	58	256.24	C_16_H_32_O_2_
25.536	Hexadecanoic acid, 15-methyl-, methyl ester; Heptadecanoic acid, methyl ester	58	284.272	C_18_H_36_O_2_

^1^ The volatile compounds were identified by GC-MS; RT: retention time; MW: molecular weight.

**Table 4:** Effect of experimental dietary treatments on average body weight (ABW) of broiler chickens at starter period.

	Age (Day)				
Treatment ^1^	1	7	10	14	21
NC	45.70 ^2^	141.15	224.00 ^b^	375.36 ^b^	780.47 ^b^
PC	45.67	150.76	246.57 ^a^	410.87 ^a^	844.13 ^a^
2000 CN	45.80	147.00	238.20 ^ab^	410.60 ^a^	850.67 ^a^
4000 CN	45.63	139.26	225.48 ^b^	374.36 ^b^	777.53 ^b^
6000 CN	45.67	141.83	226.48 ^b^	387.16 ^ab^	791.51 ^b^
Mean CN	45.70	142.70	230.05	390.71	806.57
SEM ^3^	0.029	1.471	2.709	5.020	10.604
*p*-value	0.3625	0.1022	0.0399	0.0036	0.0053

^1^ NC: negative control group = commercial feed, no additives by cinnamon powder; PC: positive control group = commercial feed, treated by antibiotic Colimox powder (mixture of amoxicillin and colistin at 200 and 150 mg/kg, respectively); 2000, 4000, and 6000 mg/kg treated groups = commercial feed, treated by cinnamon powder; ^2^ Mean of eight replicates per treatment; ^3^ SEM, standard error of mean for treatments effect; ^a,b^ mean values within columns with different superscripts are significantly different (*p* < 0.05).

**Table 5:** Effects of dietary cinnamon powder on the performance (BWG, AFI, FCR, and FE) of broiler chickens during the starter period (1–21 days of age).

Parameters	Treatment ^1^						
Period (days)	NC	PC	2000CN	4000 CN	6000 CN	SEM ^3^	*p*-Value
BWG (g)							
1–7	13.63 ^2ab^	15.01 ^a^	14.46 ^ab^	13.38 ^b^	13.74 ^ab^	0.21	0.104
1–10	20.63 ^b^	23.48 ^a^	22.43 ^ab^	21.06 ^b^	20.98 ^b^	0.328	0.037
1–14	26.36 ^2b^	29.34 ^a^	29.32 ^a^	26.44 ^b^	27.37 ^ab^	0.415	0.004
1–21	34.24 ^b^	37.48 ^a^	37.71 ^a^	34.23 ^b^	34.97 ^b^	0.501	0.003
AFI (g)							
1–7	20.58	21.4	19.7	20.4	20.08	0.254	0.196
1–10	20.3	21.13	19.49	21.2	19.48	0.365	0.384
1–14	23.84	24.75	24.28	24.41	22.95	0.389	0.539
1–21	36.04	38.32	36.53	36.9	35.82	0.474	0.3
FCR (g/g)							
1–7	1.51	1.43	1.37	1.53	1.49	0.026	0.245
1–10	0.99	0.9	0.87	1.02	0.94	0.023	0.246
1–14	0.91	0.85	0.83	0.93	0.85	0.015	0.131
1–21	1.05 ^a^	1.03 ^ab^	0.97 ^b^	1.08 ^a^	1.03 ^ab^	0.012	0.027
FE (g/g)							
1–7	0.66	0.7	0.74	0.66	0.69	0.011	0.18
1–10	1.03	1.12	1.16	1.02	1.08	0.023	0.298
1–14	1.11	1.19	1.23	1.09	1.19	0.021	0.134
1–21	0.95 ^b^	0.98 ^ab^	1.04 ^a^	0.93 ^b^	0.98 ^ab^	0.011	0.026

^1^ NC: negative control group = commercial feed, no additives by cinnamon powder; PC: positive control group = commercial feed, treated by antibiotic Colimox powder (mixture of amoxicillin and colistin at 200 and 150 mg/kg, respectively); 2000, 4000, and 6000 mg/kg treated groups = commercial feed, treated by cinnamon powder; ^2^ mean of eight replicates per treatment; ^3^ SEM, standard error of mean for treatments effect; ^a,b^ mean values within rows with different superscripts are significantly different (*p* < 0.05); BWG: body weight gain; AFI: average feed intake; FCR: feed conversion ratio; and FE: feed efficiency.

**Table 6:** Effects of dietary cinnamon bark meal (CN) on serum biochemistry (mg/dL) indices of broiler chickens at 10 days of age.

Treatment ^1^	Uric Acid	T3	T4	AST	ALT
NC	2.71 ^2^	3.79	4.92	197.44	11.35
PC	3.37	3.92	3.49	203.49	9.81
2000 CN	2.74	4.09	3.96	198.49	12.95
4000 CN	3.10	4.07	4.07	213.57	10.10
6000 CN	2.90	3.92	4.86	235.59	9.96
SEM ^3^	0.1707	0.1247	0.2287	12.273	0.463
*p*-value	0.7408	0.9472	0.6012	0.8042	0.1538

^1^ NC: negative control group = commercial feed, no additives by cinnamon powder; PC: positive control group = commercial feed, treated by antibiotic Colimox powder (mixture of amoxicillin and colistin at 200 and 150 mg/kg, respectively); 2000, 4000, and 6000 mg/kg treated groups = commercial feed, treated by cinnamon powder; ^2^ Mean of 8 chicks per treatment; ^3^ SEM, standard error of mean for treatments effect; T3: triiodothyronine; T4: thyroxine; AST = aspartate aminotransferase; ALT = alanine aminotransferase.

**Table 7:** Effect of dietary cinnamon bark meal (CN) on the relative weight (g/100 g of live BW) of internal organs, breast, and whole leg meat of female broiler chickens on 10 days of age.

Groups ^1^			Cinnamon (mg/kg)				
Parameters	NC	PC	2000	4000	6000	SEM ^2^	*p*-Value
Pancreas	0.451 ^3^	0.43	0.4	0.41	0.44	0.014	0.838
Heart	0.68	0.65	0.66	0.66	0.72	0.015	0.58
Liver	2.85	2.76	2.87	3.04	2.88	0.052	0.583
Proventriculus	0.86	0.81	0.89	0.88	0.89	0.016	0.47
Gizzard	3.13	2.87	2.96	3.03	2.91	0.041	0.254
Bursa	0.21	0.26	0.23	0.2	0.26	0.0102	0.287
Spleen	0.06	0.08	0.06	0.06	0.08	0.005	0.401
Thymus	0.41	0.51	0.45	0.53	0.56	0.024	0.192
Breast	15.65 ^b^	18.37 ^a^	19.05 ^a^	18.08 ^a^	18.67 ^a^	0.329	0.011
Legs	16.23	16.37	16.89	15.94	16.13	0.21	0.745

^1^ NC: negative control group = commercial feed, no additives by cinnamon powder; PC: positive control group = commercial feed, treated by antibiotic Colimox powder (mixture of amoxicillin and colistin at 200 and 150 mg/kg, respectively); levels 2000, 4000, and 6000 mg/kg treated groups = commercial feed, treated by cinnamon powder; ^2^ SEM, standard error of mean for treatment effect. ^3^ Data are mean of eight chicks per treatment (one bird/replicate). ^a,b^ Mean values within rows with different superscripts are significantly different (*p* < 0.05).

**Table 8:** Effects of cinnamon bark meal (CN) on the duodenal architecture of female broiler chickens at 10 days of age.

Groups			Cinnamon (mg/kg)				
Treatment ^1^	NC	PC	2000	4000	6000	SEM ^3^	*p*-Value
VL (μm)	552.47 ^2bc^	537.34 ^c^	648.14 ^a^	610.27 ^ab^	585.10 ^abc^	11.4625	0.0098
VW (μm)	110.93 ^a^	85.70 ^b^	81.35 ^b^	91.77 ^b^	87.02 ^b^	2.2766	<0.0001
VSA (mm^2^)	0.19 ^a^	0.15 ^b^	0.17 ^ab^	0.17 ^ab^	0.16 ^b^	0.0046	0.0092
CD (μm)	58.52 ^b^	67.38 ^ab^	57.60 ^b^	69.52 ^ab^	72.28 ^a^	1.911	0.0393
VL/CD	9.25 ^b^	8.17 ^b^	13.48 ^a^	7.54 ^b^	8.14 ^b^	0.6445	0.0239
GCD	64.80 ^d^	66.27 ^d^	102.44 ^a^	79.00 ^c^	91.42 ^b^	2.4996	<0.0001
GCD/100 µm VA	5.81 ^b^	6.18 ^b^	8.03 ^a^	6.53 ^b^	7.88 ^a^	0.1986	<0.0001

^1^ NC: negative control group = commercial feed, no additives by cinnamon powder; PC: positive control group = commercial feed, treated by antibiotic Colimox powder (mixture of amoxicillin and colistin at 200 and 150 mg/kg, respectively); 2000, 4000, and 6000 mg/kg treated groups = commercial feed, treated by cinnamon powder. ^2^ Data are mean of 10 well-oriented villi. ^3^ SEM, standard error of mean for treatment effect. ^a–d^ Mean values within rows with different superscripts are significantly different (*p* < 0.05). VL: villus length; VW: villus width; VSA: villus surface area; CD: crypt depth; VL/CD: villus length:crypt depth; GCD: goblet cell density, goblet cell density per 100 μm villus area (GCD/100 µm VA).

**Table 9:** Effect of cinnamon bark meal (CN) on the cecal microflora (log colony-forming units ^*^/g digesta) of female birds at 10 days of age.

Treatment ^1^	*Lactobacillus*	Aerobic	*Escherichia coli*	*Salmonella*	*Lactobacillus*/*Escherichia coli*
NC	6.65 ^2^	8.795	7.32 ^c^	3.39	0.92 ^a^
PC	5.34	9.451	7.82 ^bc^	3.48	0.73 ^ab^
2000 CN	6.01	9.810	11.41 ^a^	3.82	0.53 ^b^
4000 CN	6.17	9.404	10.70 ^a^	3.84	0.58 ^b^
6000 CN	5.89	9.154	9.90 ^ab^	4.33	0.60 ^b^
SEM ^3^	0.156	0.446	0.514	0.563	0.047
*p*-value	0.085	0.978	0.012	0.182	0.022

^1^ NC: negative control group = commercial feed, no additives by cinnamon powder; PC: positive control group = commercial feed, treated by antibiotic Colimox powder (mixture of amoxicillin and colistin at 200 and 150 mg/kg, respectively); 2000, 4000, and 6000 mg/kg treated groups = commercial feed, treated by cinnamon powder. ^2^ Mean of eight replicates per treatment. ^3^ SEM, standard error of mean for treatment effect. ^a–c^ Mean values within columns with different superscripts are significantly different (*p* < 0.05).

## Data Availability

All data sets collected and analyzed during the current study are available from the corresponding author on fair request.
